# Comparison on Phenolic Compounds and Antioxidant Properties of Cabernet Sauvignon and Merlot Wines from Four Wine Grape-Growing Regions in China

**DOI:** 10.3390/molecules17088804

**Published:** 2012-07-25

**Authors:** Bao Jiang, Zhen-Wen Zhang

**Affiliations:** 1 College of Enology, Northwest A&F University, Yangling 712100, Shaanxi, China; Email: treebaojiang@163.com; 2 Weinan Vocational & Technical College, Weinan City 714000, Shaanxi, China; 3 Shaanxi Engineering Research Center for Viti-Viniculture, Northwest A&F University, Yangling 712100, Shaanxi, China

**Keywords:** wines, phenolic compounds, antioxidant activity, wine grape-growing regions

## Abstract

The antioxidant activities in the Cabernet Sauvignon and Merlot wines from four wine grape-growing regions in China were measured by different analytical assays: 2,2-diphenyl-1-picrylhydrazyl (DPPH·), cupric reducing antioxidant capacity (CUPRAC), superoxide radical-scavenging activity (SRSA) and the contents of total phenols, total flavonoids, total flavanols and total anthocyanins were determined. The results showed that the contents of phenolic compounds and the levels of antioxidant activity in the wine samples greatly varied with cultivar and environmental factors of vine growth. The contents of phenolic compounds and antioxidant activities in Cabernet Sauvignon and Merlot wines from the Yuquanying region of Ningxia were significantly higher than other three regions, followed by the wines from Shacheng region of Hebei, and these parameters were the lowest in Cabernet Sauvignon and Merlot wines from the Changli regions of Hebei and Xiangning region of Shanxi. Taken together, a close relationship between phenolic subclasses and antioxidant activity was observed for the wine samples. Moreover, there were significant discrepancies in the individual phenolic composition and content of four regional Cabernet Sauvignon and Merlot wines, among which the individual phenolic compounds (catechin, epicatechin, cinnamic acid, quercetin-3-*O*-glucuronide, quercetin-3-*O*-glucoside, laricitrin-3-*O*-glucoside and isorhamnetin-3-*O*-glucoside) revealed a significant correlation (*p* < 0.05) with the antioxidant capacity in present study, especially for catechin and epicatechin.

## 1. Introduction

Phenolic compounds, which are abundant in grape berries and wines, play one of the most important roles in the quality of grape berries and wines. They strongly contribute to the color, mouth feel and palatability of red wines [1], moreover, polyphenols also exert many favorable effects on human health, such as the inhibition of atherosclerosis, coronary heart disease and various cancer types [2,3]. Phenolic compounds can be commonly classified into five groups: anthocyanins, flavan-3-ols, flavonols, stilbene derivatives and phenolic acids (including hydroxybenzoic acids and hydroxycinnamic acids) which are considered to possess the ability to scavenge excess radicals and maintain the balance of reactive oxygen species (ROS) in the human body. Positive correlations between total phenolics and antioxidant capacity have been reported [4,5]. According to many authors, antioxidant activity of grape berries and wines results mainly from their phenolics, whereas the phenolic content and composition depend on the grape variety, vineyard location, cultivation system, climate, soil types, vine cultivation practices, harvesting time, production process and ageing [6].

The antioxidant compositions of samples are fairly complex, usually involving multiple reaction characteristics and mechanisms, so no single assay will accurately reflect all antioxidants in a mixed or complex system, therefore different antioxidant capacity assays may be needed.

The wine industry in China is growing, the wine market has a great space to develop, and to date, several wine grape-growing regions have been developed, but unlike other counties in the World whose wine grape-growing regions are relatively centralized, China has a very scattered distribution of wine grape-growing regions whose ecological conditions are significantly different, with a distance of over 2,000 km either from south to north or from east to west [7]. In addition, the wine grape-growing regions of China are display unique ecological conditions: the Ningxia Helan Zone is beyond the mountains; the Xiangning Shanxi Zone is located on Loess Plateau region where the characteristic landform feature are crisscross gulleys; Changli Hebei Zone is on the seaside and Shacheng Hebei Zone is located in the basin. All the climate and soil characteristics of China’s wine grape-growing regions can influence the wine grapes’ quality to a different extent. Although a great difference in climate and soil conditions exists in China’s wine grape-growing regions, both “Cabernet Sauvignon and Merlot” which are well-known *Vitis vinifera* cultivars, are still the main cultivars in each region because of their strong adaptability and premium quality wines. However, the differences between phenolic compounds and antioxidant activities of Cabernet Sauvignon and Merlot wines from four wine grape-growing regions in China remains unclear.

In the present study, the tested Cabernet Sauvignon and Merlot wines were from four different wine grape-growing regions in China, and this study aimed to analyze the differences in the phenolic compounds and antioxidant properties of these samples. The objectives were to evaluate the levels of phenolic compound and antioxidant activities of these samples and provide some useful information for producing high-quality wine in China. 

## 2. Results and Discussion

### 2.1. Comparison on Phenolic Compounds

The total phenols (TP), total flavonoids (TFO), total flavanols (TFA) and total anthocyanins (TA) were measured for all the wine samples from the four regions, and the results are shown in Table 1. There was a wide range of phenol concentrations in wine samples tested.

**Table 1:** Total amount of phenolic substances of Cabernet Sauvignon and Merlot wines from four different wine grape-growing regions.

Analytical Index	Cabernet Sauvignon				Merlot			
	NXYQY	SXXN	HBCL	HBSC	NXYQY	SXXN	HBCL	HBSC
TP ^a^ (GAE)	2710.4 ± 200.5a	1129.8 ± 79.6c	1313.0 ± 45.2c	2330.2 ± 120.3b	1656.5 ± 161.7a	860.2 ± 45.7c	941.2 ± 66.4c	1247.7 ± 110.1b
TFO ^b^ (CTE)	2290.3 ± 157.2a	859.9 ± 55.5d	1189.1 ± 89.1c	1906.4 ± 132.9b	1375.4 ± 76.4a	697.5 ± 23.1c	660.6 ± 18.5c	1031.4 ± 95.4b
TFA ^b^ (CTE)	532.3 ± 79.8b	277.7 ± 34.3c	342.7 ± 47.6c	666.4 ± 84.6a	497.4 ± 16.4a	279.8 ± 24.1c	272.8 ± 40.0c	361.1 ± 10.3b
TA ^c^	400.3 ± 16.3a	286.8 ± 27.9b	261.5 ± 42.5b	372.6 ± 4.2a	350.3 ± 54.8a	259.4 ± 0.8b	157.5 ± 17.5c	216.7 ± 20.8bc

Different letters within the same column for the same cultivar indicate significant difference at *p* *<* 0*.*05 (Duncan’s test); ^a^ Total phenolics (TP) of wines expressed as milligrams of gallic acid equivalents per liter basis (mg GAE/L); ^b^ Total flavonoids (TFO) of wines expressed as milligrams of catechin equivalents per liter basis (mg CTE/L), expression method of TFO and total flavanols (TFA) were identical; ^c^ Total anthocyanins (TA) of wines expressed as milligrams of malvidin-3-*O*-glucoside equivalents (ME) per liter basis (ME mg/L).

#### 2.1.1. Total Phenols

The TP contents varied from 1,129.8 to 2,710.4 mg GAE/L, for the Cabernet Sauvignon wines and from 860.2 to 1,656.5 mg GAE/L, for the Merlot wines. The Cabernet Sauvignon and Merlot wines with the highest amount of TP were both from the NXYQY region, whereas both single varietal wine samples tested in the SXXN region had the lowest values. The content of TP decreased in the order: NXYQY > HBSC > HBCL > SXXN regions for Cabernet Sauvignon and Merlot wines. For Cabernet Sauvignon samples, the TP content in NXYQY region was nearly 2.0~2.4 times those in the SXXN and HBCL regions, being approximately 1.2 times that in the HBSC region. For Merlot samples, the content of TP in the NXYQY region was 1.7~1.9 times those in the SXXN and HBCL regions, whereas the TP content in the HBCL region was non-significantly higher than that in the SXXN region. 

#### 2.1.2. Total Flavonoids

The content of TFO varied from 859.9 to 2,290.3 mg CTE/L, for the Cabernet Sauvignon wines and from 660.6 to 1,375.4 mg GAE/L, for the Merlot wines. The TFO contents of Cabernet Sauvignon and Merlot wines in the NXYQY region were significantly higher than those of the other three regions. In addition, the Merlot wines from the HBCL and SXXN regions contained non-significantly lower content of TFO. The content of TFO decreased in the order: NXYQY > HBSC > HBCL > SXXN for Cabernet Sauvignon wines and NXYQY > HBSC > SXXN > HBCL for Merlot wines, respectively.

#### 2.1.3. Total Flavanols

The content of TFA varied from 277.7 to 666.4 mg CTE/L, for the Cabernet Sauvignon wines and from 272.8 to 497.4 mg GAE/L, for the Merlot wines. The TFA content of Cabernet Sauvignon wines in the NXYQY and HBSC regions were more than double that in the SXXN region, and its content in Merlot samples in the NXYQY region was more than 70% those in the HBCL and SXXN regions. The content of TFA decreased in the order: HBSC > NXYQY > HBCL > SXXN (Cabernet Sauvignon wines) and NXYQY > HBSC > SXXN >HBCL (Merlot wines), respectively.

#### 2.1.4. Total Anthocyanins

The grape pigments or anthocyanins are present in red grapes only and are largely responsible for the color of red wines. The content of TA varied from 261.5 to 400.3 ME mg/L, for the Cabernet Sauvignon wines and from 157.5 to 350.3 ME mg/L, for the Merlot wines. The TA contents of two single varietal wines in the NXYQY region were the highest, and the contents in the HBCL region were the lowest. In order to identify the phenolic contents of Cabernet Sauvignon and Merlot wines from four wine grape-growing regions, we chose all the raw materials (grape berries) and wines had the same situations, including the same vintage and cultivation management, the same winemaking techniques and ageing. The results confirm a variation in phenolic content among wine samples tested. As is well known, the amounts of phenolic materials vary considerably in different wine grape-growing regions, depending on the grape variety, environmental factors of vine growth [8]. On the whole, in the present study, either for Cabernet Sauvignon or for Merlot, the NXYQY wine samples contained significantly more contents of phenolics than did the other regional wines, while on the contrary, its contents in SXXN and HBCL regions were the lowest. The high phenolic contents indicated that the NXYQY wines had potential antioxidant activity. In the study of Li *et al.* [9], the TP contents of Cabernet Sauvignon and Merlot wines were reported. The TP content varied from 1,580 to 2,927 mg CTE/L for the Cabernet Sauvignon wines, its content in the Merlot wines varied from 1,977 to 2,246 mg GAE/L. Considering the fact that there exist differences of environmental conditions, winemaking techniques and aging, these values are analogous to the values found in this study for Cabernet Sauvignon and Merlot wines.

### 2.2. Comparison on Antioxidant Activity

**Figure 1:**
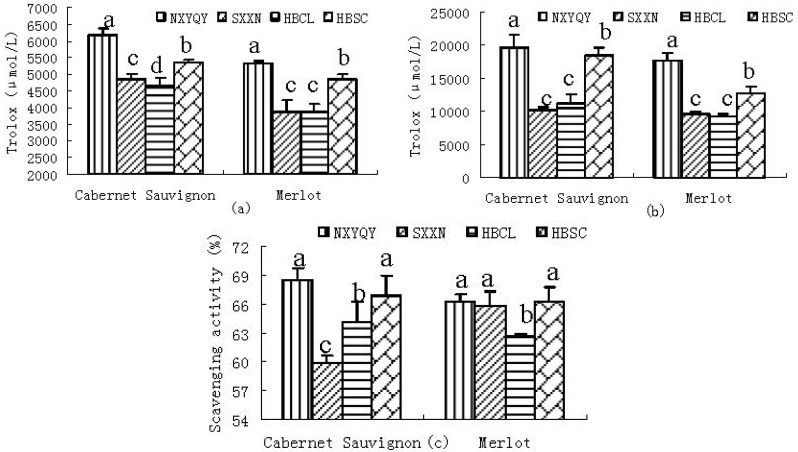
Antioxidant activity of the extracts of Cabernet Sauvignon and Merlot grape berries from four different wine grape-growing regions, determined using three kinds of assays. (**a**) 2,2-Diphenyl-1-picrylhydrazyl (DPPH) radical (DPPH•) scavenging activity of samples; (**b**) Cupric ion reducing antioxidant capacity (CUPRAC) of samples; and (**c**) Superoxide radical (O_2_^−^) scavenging activity (SRSA) of samples. Data are expressed as mean ± standard deviation. Different letters above the bars for the same cultivars indicate significant difference at *p* *<* 0*.*05 (Duncan’s test).

The antioxidant activities found by three different assays in the wines differed significantly (Figure 1). As can be observed, the values of Cabernet Sauvignon and Merlot wines with the highest polyphenol contents in the NXYQY region were higher than those of the other regional samples in every antioxidant test used. The magnitude of the difference depends on the method employed. This result is well in accordance with recent reports in the literature [5,6], suggesting a positive correlation between phenolic compound contents and antioxidant capacity.

#### 2.2.1. DPPH• Scavenging Activity

Analysis of DPPH• scavenging activity is one method for detecting antioxidant activity and is commonly used to evaluate the ability of free radicals to react with antioxidants [10,11]. In the DPPH• scavenging assay, antioxidants reacting with DPPH• produce yellow *α*,*α*-diphenyl-*β*-picrylhydrazine. The degree of discoloration indicates the radical scavenging activity of the antioxidant [12]. The free radical-scavenging activity of all the samples was determined by the DPPH• methods and the results were shown in Figure 1(a). For DPPH•, the values of wines varied from 4,670.4 to 6,179.4 μM TE/L for the Cabernet Sauvignon wines, and 3,857.0 to 5,304.1 μM TE/L for the Merlot wines. The values of DPPH• decreased in the order: XNYQY > HBSC > SXXN > HBCL (Cabernet Sauvignon) wines and XNYQY > HBSC > HBCL > SXXN (Merlot wines), respectively. The results of investigation show that the higher the concentration of antioxidant, the lower is the amount of remaining DPPH• and the higher is the free radical-scavenging activity.

#### 2.2.2. CUPRAC

The reducing power property indicates that the antioxidant compounds are electron donors and can reduce the oxidized intermediates of the lipid peroxidation process. In this research, we use the CUPRAC assay which is based on the reduction of Cu(II) to Cu(I) by antioxidants. This assay is superior to the widely used ferric ion reducing antioxidant power (FRAP) assay. Firstly, the reaction conditions are more adaptable to real body systems; secondly, it can detect the reducing ability of thiol compounds [9]. All analyzed wine samples demonstrated significant antioxidant capacity in the CUPRAC test [Figure 1(b)]. For CUPRAC, the values of wines varied from 10,227.2 to 19,632.8 μM TE/L for the Cabernet Sauvignon wines, and 9,194.1 to 17,693.0 μM TE/L for the Merlot wines. The CUPRAC of the NXYQY Cabernet Sauvignon and Merlot wines with the highest values was nearly 1.9 times higher than those of the SXXN Cabernet Sauvignon and the HBCL Merlot wine, respectively. Not significant differences were also observed in the SXXN and HBCL Merlot wines. The values of CUPRAC decreased in the order: NXYQY > HBSC > HBCL > SXXN Cabernet Sauvignon wines and XNYQY > HBSC > SXXN > HBCL Merlot wines.

#### 2.2.3. SRSA

The superoxide radical is one extremely reactive free radical formed in biological systems and has been implicated as a highly damaging species in free radical pathology, capable of damaging almost every molecule found in living cells [13]. The superoxide radical-scavenging activities of all the wine samples were shown in Figure 1(c). In this research, the Cabernet Sauvignon and Merlot samples exhibited from 59.9% to 68.5% and 62.6% to 66.2% superoxide radical-scavenging activity, respectively. The inhibition of superoxide radical decreased in the order: XNYQY > HBSC > HBCL > SXXN (Cabernet Sauvignon) wines and XNYQY and HBSC > SXXN > HBCL (Merlot wines). 

### 2.3. Comparison on Individual Phenolics

A total of 24 and 26 non-anthocyanins phenolic compounds (NAPC) were identified from “Cabernet Sauvignon” and “Merlot” wine samples using HPLC-ESI-MS/MS (Table 2), respectively, including five flavan-3-ols, 13 flavonols, seven phenolic acids, and one stilbene. The results obtained confirm a variation in the NAPC composition and content amongst wines tested, due to their different environmental factors in the vineyard, indicating that NAPC are subject to the impact of environmental factors.

#### 2.3.1. Comparison on Flavan-3-ols

Five flavan-3-ols were observed in four regional wines by HPLC-ESI-MS/MS (Table 2), including three monomers (catechin, epicatechin and gallocatechin) and two dimers. With the exception of gallocatechin, which was only detected in the NXYQY Cabernet Sauvignon wines and the HBSC Merlot wines, the wine samples from four regions contained the same composition of four flavan-3-ols. For the Cabernet Sauvignon wines, the wine sample with the highest content in five flavan-3-ols was that from the NXYQY region, except for procyanidin dimer B2, and the wine from SXXN region showed the lowest levels of five flavan-3-ols. The contents of five flavan-3-ols in HBSC and HBCL regional wines varied among these regions, being comparable. In more detail, the contents of catechin, epicatechin and procyanidin dimer B2 in the NXYQY “Cabernet Sauvignon” wines were almost 8, 14 and 34 times those in the wines from the SXXN region. 

**Table 2:** Concentrations of individual phenolic compounds of Cabernet Sauvignon and Merlot wines from four different wine grape-growing regions (mg /L).

Compound	Cabernet Sauvignon Wine				Merlot Wine				Retention Time (Min)	Molecular Ions (M^+^)	Fragment Ions (MS^2^, *m/z*)
	NXYQY	SXXN	HBSC	HBCL	NXYQY	SXXN	HBSC	HBCL			
Flavan-3-ols											
Catechin	62.4 ± 3.7	8.1 ± 0.8	48.2 ± 9.6	45.2 ± 3.5	23.2 ± 3.5	15.4 ± 2.1	29.5 ± 2.0	14.9 ± 4.8	3.14	289	245, 205
Epicatechin	81.8 ± 11.7	6.0 ± 0.1	40.5 ± 4.7	31.5 ± 2.3	32.2 ± 0.7	16.8 ± 2.4	42.7 ± 5.9	27.1 ± 2.8	6.12	289	245
Gallocatechin	1.9 ± 0.1	—	—	—	—	—	1.0 ± 0.1	—	0.36	305	179, 125
Procyanidin dimer B2	54.7 ± 5.8	14.0 ± 1.3	14.9 ± 1.7	7.6 ± 0.6	22.7 ± 4.4	7.8 ± 1.4	5.7 ± 0.4	25.1 ± 1.3	2.32	577	425, 289
Procyanidin dimer B3	55.7 ± 4.1	1.6 ± 0.6	7.6 ± 0.8	9.4 ± 0.8	5.3 ± 0.6	2.2 ± 0.3	7.8 ± 0.4	23.7 ± 1.4	3.27	865	695, 577, 287
Flavonols											
Myricetin-3-*O*-galactoside	2.2 ± 0.1	3.0 ± 0.2	5.6 ± 0.6	1.6 ± 0.1	—	4.8 ± 0.4	0.9 ± 0.0	3.9 ± 0.4	13.34	479	317
Myricetin-3-*O*-glucoside	4.0 ± 0.6	2.2 ± 0.3	—	4.1 ± 0.8	5.0 ± 0.7	6.0 ± 0.3	6.8 ± 0.1	5.5 ± 0.3	14.12	479	317, 179
Myricetin-3-*O*-glucuronide	—	—	1.4 ± 0.1	—	—	—	trace	—	13.57	493	317
Quercetin-3-*O*-hexoside	2.9 ± 0.4	1.5 ± 0.3	—	—	—	—	9.0 ± 0.7	—	4.43	463	301
Quercetin-3-*O*-galactoside	6.4 ± 0.4	5.5 ± 0.3	—	3.1 ± 0.0	7.1 ± 1.4	5.1 ± 0.6	6.5 ± 0.5	—	19.15	463	301
Quercetin-3-*O*-glucuronide	11.0 ± 0.3	1.4 ± 0.1	4.6 ± 0.4	4.8 ± 0.3	11.9 ± 0.6	4.1 ± 0.3	4.0 ± 0.3	4.0 ± 0.4	19.68	477	301
Quercetin-3-*O*-glucoside	—	2.1 ± 0.1	—	3.8 ± 0.4	—	2.6 ± 0.2	5.3 ± 0.8	—	20.84	463	301
Dihydroquercetin-3-*O*-rhamnoside	10.5 ± 0.7	2.7 ± 0.3	5.4 ± 0.4	6.6 ± 0.6	8.9 ± 0.6	8.1 ± 0.3	1.1 ± 0.0	7.1 ± 0.3	18.84	449	285, 151
Laricitrin-3-*O*-glucoside	13.7 ± 0.6	0.9 ± 0.1	5.0 ± 0.7	6.0 ± 0.4	8.2 ± 0.4	1.9 ± 0.1	5.0 ± 0.3	5.5 ± 0.6	23.13	493	331
Kaempferol-3-*O*-galactoside	5.2 ± 0.3	1.5 ± 0.1	—	2.3 ± 0.0	—	2.3 ± 0.1	5.1 ± 0.4	3.5 ± 0.3	23.57	447	285
Kaempferol-3-*O*-glucoside	—	2.3 ± 0.3	—	4.3 ± 0.4	4.8 ± 0.4	2.7 ± 0.2	—	—	24.08	447	285
Isorhamnetin-3-*O*-glucoside	9.6 ± 0.3	1.6 ± 0.1	—	2.8 ± 0.3	4.5 ± 0.6	2.3 ± 0.0	2.1 ± 0.1	—	29.66	477	314
Isorhamnetin-3-*O*-galactoside	—	3.0 ± 0.4	—	—	—	—	4.3 ± 0.6	—	7.45	477	315
Hydroxybenzoic acids											
DEGCEC ^a^	0.3 ± 0.0	0.4 ± 0.1	0.2 ± 0.0	trace	0.2 ± 0.1	0.5 ± 0.1	0.1 ± 0.0	1.4 ± 0.3	0.36	593	425, 289, 407
Hx-es-va ^b^	—	—	—	—	—	—	2.1 ± 0.1	—	4.94	329	167, 191
Syringic acid	5.2 ± 0.4	0.8 ± 0.2	1.5 ± 0.1	3.8 ± 0.4	5.0 ± 0.6	0.3 ± 0.0	0.9 ± 0.1	11.3 ± 1.1	8.07	197	182, 153
Hx-es-pc-a ^c^	1.9 ± 0.4	—	—	0.8 ± 0.0	—	—	4.7 ± 0.5	—	1.81	315	153
Hydroxycinnamic acids											
Cinnamic acid	1.7 ± 0.3	0.9 ± 0.2	—	0.9 ± 0.1	1.4 ± 0.0	0.8 ± 0.1	—	1.2 ± 0.2	38.90	147	
trans-Fertaric acid	—	0.9 ± 0.0	1.6 ± 0.1	0.9 ± 0.2	4.3 ± 0.3	1.1 ± 0.2	—	0.7 ± 0.1	3.04	325	193
Hx-es-fa ^d^	—	—	—	—	—	—	0.9 ± 0.1	—	1.27	355	193
Stilbenes											
trans-Piceid	—	0.5 ± 0.0	11.6 ± 0.8	—	1.5 ± 0.2	4.3 ± 0.4	7.5 ± 0.8	1.8 ± 0.3	24.83	389	227

“—”, not detected; the results were average of two injections; ^a^ DEGCEC, dimer(epi)gallocatechin-(epi)catechin; ^b^ Hx-es-va, hexose ester of vanillic acid; ^c^ Hx-es-pc-a, hexose ester of protocatechuic acid; ^d^ Hx-es-fa, hexose ester of ferulic acid.

For the Merlot wines, the HBSC regional wine had the highest level of catechin and epicatechin, which were almost two times that in the wines from the SXXN and HBCL region, and nearly three times that in the SXXN wine sample. The interregional differences were also present in the contents of the procyanidin dimers.

#### 2.3.2. Comparison of Flavonols

Flavonols are yellow pigments that mainly exist as the 3-*O*-glycoside of four main aglycones: myricein, quercetin, kaempherol and isorhamnetin [14]. They generally act as UV protectors [15,16] and co-pigments of anthocyanins in flowers and fruits [17,18]. As shown in Table 2, there were 13 flavonols identified in all wines, and the composition of flavonols varied widely with geographical origin. Compared to the other three regions, the number of flavonols detected in HBSC Cabernet Sauvignon and HBCL Merlot wines was the least, respectively. Only quercetin-glucuronide, dihydroquercetin-3-*O*-rhamnoside, laricitrin-3-*O*-glucoside and myricetin-3-*O*-galactoside were common in the Cabernet Sauvignon wines of four regions, besides including the former three flavonols, myricetin-3-*O-*glucoside was common in all the Merlot wines. The greatest range of flavonol compounds was found in the NXYQY wines. Except for myricetin-3-*O*-galactoside and common in the Cabernet Sauvignon wines of four regions, besides including former three flavonols, myricetin-3-*O-*glucoside was common in all the Merlot wines. The greatest range of flavonol compounds was found in the NXYQY wines. Except for myricetin-3-*O*-galactoside and myricetin-3-*O-*glucoside, the contents of other flavonols were the highest in the NXYQY region, as shown in Table 2.

#### 2.3.3. Comparison on Phenolic Acids

The phenolic acids could be simply classified into two groups: hydroxybenzoic acid and hydroxycinnamic acid. There were four hydroxybenzoic acids and three hydroxycinnamic acid identified in all wines (Table 2). As shown by this data, the contents and composition of phenolic acids varied widely in wine samples of different regions. Only dimer(*epi*)gallocatechin-(*epi*)catechin and syringic acid were common in the Cabernet Sauvignon and Merlot wines of all four regions. Syringic acid is one of the most important hydroxybenzoic acids in wines. Its contents showed significant differences in the wines from different regions. The NXYQY wines had the highest level of syringic acid while the wines from SXXN presented the lowest level. Among various hydroxycinnamic acids, the content of *trans*-fertaric acid showed significant differences in the different regions. The NXYQY Cabernet Sauvignon wines had nearly six times more *trans*-fertaric acid than the HBCL wines.

#### 2.3.4. Comparison on Stilbene

Since stilbenes are phytoalexins and their existence in grapes is directly related to environmental stress, such as botrytis infections and UV-irradiation [19], healthy plants contain small amounts. The *trans*-piceid was only found in all Merlot wine samples, amounts were comparable with the concentration range found in the literature [8]. Wine samples from the HBSC region have the highest amount of *trans*-piceid.

The phenol profile in a wine comes primarily from the grape berries, but the polyphenolic composition of wines is more complex as compared to their corresponding grape berries, because of the numerous reactions involving phenolic compounds that occur during the wine making and maturation processes [14]. In the present study, we chose all the raw materials (cultivation management) and wines (winemaking techniques and ageing) to have the same situations, yet the four regional wines displayed different qualitative and quantitative profiles, attributed to the unique soil and climatic conditions of the four regions and are indicative of the “terroir” influence on a wine’s polyphenolic profile. It must be noted that these individual phenols contents with different composition were all under 10.0 mg/L. The results were partially inconsistent with the results in some previous studies [8,20] showing that a similar composition in phenolic compounds was observed in different regional wines due to the biosyntheses of phenolic compounds depending largely on the genotypes of the grape cultivar rather than the environmental factors. Downey *et al.* [21] also suggested that flavonoid biosynthesis in plants is affected by many factors, including light, temperature, altitude, soil type, water, microbial interactions and nutritional status. Our finding was in accordance with these studies on polyphenolic composition involving wines of different geographical origins [22]. The NXYQY wines had the highest level of individual phenols, especially for four flavan-3-ols and quercetin-3-*O*-glucuronide, dihydroquercetin-3-*O*-rhamnoside, laricitrin-3-*O*-glucoside and isorhamnetin-3-*O*-glucoside. Price *et al.* [23] and Spayd *et al.* [24] have suggested that there was a positive correlation between sunlight exposure and an increase in flavonols. Previous researches showed that light, water deficits and higher temperature differences between daytime and nighttime could significantly increase the contents of flavonoids [25,26]. In addition, both the HBSC and HBCL regions that are geographically close to each other have similar individual phenol contents in Cabernet Sauvignon wines.

### 2.4. Correlation

Correlation analysis was used to explore the relationships amongst the different antioxidant variables measured for all the wine samples (Table 3). The total phenols, flavonoids, flavanols and anthocyanins contents of wine samples almost exhibited the strongest correlation (*p* < 0.01 or *p* < 0.05) with antioxidant properties, except that polyphenolic compounds exhibited weaker correlation with SRSA assay. 

**Table 3:** Pearson’s correlation coefficients of antioxidant capacity, TP, TFO, TFA and TA in Cabernet Sauvignon and Merlot wines from four different wine grape-growing regions.

	TP	TFO	TFA	TA	DPPH	CUPRAC	SRSA
**TP**	1	0.962 **	0.967 **	0.856 **	0.875 **	0.945 **	0.641 ^ns^
**TFO**		1	0.884 **	0.865 **	0.920 **	0.933 **	0.700 ^ns^
**TFA**			1	0.811 *	0.771 *	0.938 **	0.693 ^ns^
**TA**				1	0.852 **	0.874 **	0.551 ^ns^
**DPPH**					1	0.899 **	0.537 ^ns^
**CUPRAC**						1	0.746 *
**SRSA**							1

^ns^ nonsignificant; ** Correlation is significant at the 0.01 level (2-tailed); * Correlation is significant at the 0.05 level (2-tailed).

Thus, the antioxidant efficiency of Cabernet Sauvignon and Merlot wines tested appear to be largely influenced by the total phenols, flavonoids and flavanols, these results were in agreement with previous reports in the literatures [27,28,29]. 

Amongst the methods used for quantifying antioxidant activities, the significant correlation (*p* < 0.01 or *p* < 0.05) between methods was confirmed with three methods (DPPH, CUPRAC and SRSA), the correlation between CUPRAC and DPPH, SRSA was 0.899 and 0.746, respectively. The correlation between DPPH and SRSA was only 0.537. This result suggested that CUPRAC and DPPH assays were almost comparable and interchangeable in characteristing the wine antioxidant activities. These results were also in agreement with another report in the literature [9].

In order to determine the contribution of individual phenolic compounds to the antioxidant capacity, the correlation between the antioxidant capacity estimated by the three methods and the concentration all the phenolic compounds detected was investigated (Table 4). Catechin and epicatechin among the flavan-3-ols correlated significantly (*p* < 0.05) with all three antioxidant capacity assays. This shows that both compounds can make a major contribution to the overall antioxidant power of wines. Hence, the higher antioxidant capacity exhibited by wines, especially those belonging to NXYQY and HBSC regional wines could be partially due to the relatively high concentrations of catechin and epicatechin. The high antioxidant activity of catechin and epicatechin has been demonstrated by others authors [30,31,32,33,34].

**Table 4:** Pearson’s correlation coefficients between antioxidant capacity (DPPH, CUPRAC and SRSA methods) and individual phenolic compounds in Cabernet Sauvignon and Merlot wines from four different wine grape-growing regions.

Phenolic Compounds	DPPH method	CUPRAC method	SRSA method	Phenolic Compounds	DPPH method	CUPRAC method	SRSA method
Catechin	0.730 *	0.723 *	0.715 *	Kaempferol-3-*O*-galactoside	0.539	0.710	0.752
Epicatechin	0.747 *	0.743 *	0.783 *	Kaempferol-3-*O*-glucoside	0.585	0.792	0.613
Gallocatechin	—	—	—	Isorhamnetin-3-*O*-glucoside	0.845 *	0.883 *	0.685
Procyanidin dimer B2	0.630	0.587	0.385	Isorhamnetin-3-*O*-galactoside	—	—	—
Procyanidin dimer B3	0.529	0.451	0.435	DEGCEC ^a^	−0.634	−0.585	−0.477
Myricetin-3-*O*-galactoside	−0.242	0.078	0.022	Hx-es-va ^b^	—	—	—
Myricetin-3-*O*-glucoside	−0.362	−0.081	0.505	Syringic acid	−0.120	−0.042	−0.100
Myricetin-3-*O*-glucuronide	—	—	—	Hx-es-pc-a ^c^	−0.135	−0.076	0.248
Quercetin-3-*O*-hexoside	−0.328	−0.089	0.425	Cinnamic acid	0.772	0.873 *	0.629
Quercetin-3-*O*-galactoside	0.491	0.624	0.385	trans-Fertaric acid	0.604	0.731	0.506
Quercetin-3-*O*-glucuronide	0.642	0.755 *	0.673	Hx-es-fa ^d^	-	-	-
Quercetin-3-*O*-glucoside	0.402	0.955 *	0.659	trans-Piceid	0.356	0.521	0.683
Dihydroquercetin-3-*O*-rhamnoside	0.222	0.393	0.458	Laricitrin-3-*O*-glucoside	0.725 *	0.731 *	0.671

** Correlation is significant at the 0.01 level (2-tailed); * Correlation is significant at the 0.05 level (2-tailed); ^a^ DEGCEC, dimer(*epi*)gallocatechin-(*epi*)catechin; ^b^ Hx-es-va, hexose ester of vanillic acid; ^c^ Hx-es-pc-a, hexose ester of protocatechuic acid; ^d^ Hx-es-fa, hexose ester of ferulic acid.

A positive correlation between CUPRAC assay and quercetin-3-*O*-glucuronide (r = 0.755), quercetin-3-*O*-glucoside (r = 0.955) and cinnamic acid (r = 0.873) were also observed. Besides, laricitrin-3-*O*-glucoside and isorhamnetin-3-*O*-glucoside correlated significantly (p < 0.05) with DPPH assay and CUPRAC assay, while SRSA assay exhibited weaker correlations with the two flavonols. These meant that quercetin-3-*O*-glucuronide, quercetin-3-*O*-glucoside, cinnamic acid, laricitrin-3-*O*-glucoside and isorhamnetin-3-*O*-glucoside all contributed to the antioxidant capacities of wines. This activity is due to their structure, characterized by the presence of several ortho hydroxyl substituents which exhibit a higher ability to donate a hydrogen atom and to support the unpaired electron. It is concluded that the wine’s antioxidant properties are influenced differently by each polyphenolic molecule, but further researches are necessary to confirm this hypothesis.

## 3. Experimental

### 3.1. Sample Collection and Vinification

Four wine-growing regions with distinct terroir characters were selected for sampling grapes, including the cool and semi-arid area in Western China (Yuquanying of Ningxia, henceafter NXYQY), the warm and semi-arid area in Eastern China (Shacheng of Hebei, henceafter HBSC) and the arid area in North-China (Xiangning of Shanxi, henceafter SXXN) and the wetter area in Eastern China (Changli of Hebei, henceafter HBCL) (Table 5).

**Table 5:** Grape grower, regional climate condition, and soil type from four different wine grape growing-regions.

Regions	Grape Growers (Sampling Sources)	Regional soil types	Regional Climate Conditions
Yuquanying of Ningxia, (NXYQY)	Huibin Grape Co. Ltd.	Gravelly soils	The vineyards are located on the alluvial plain at the altitude of about 1,036 m with a cool and semi-arid climate and a big temperature difference between daytime and night time; with an annual accumulated temperature being 3,298–3,351 °C; with abundant sunshine and an annual rainfall of 150–200 mm.
Xiangning of Shanxi, (SXXN)	Chateau Rongzi Co. Ltd.	Clay loamy soil	The vineyards are located on Loess Plateau of China at the average altitude of about 1,100 m with a cold and arid climate and a big temperature difference between daytime and night time; with an annual accumulated temperature being 2,998 °C; with abundant sunshine and an annual rainfall of 400–600 mm and an annual sunshine time of 2,200–2,500 h.
Changli of Hebei, (HBCL)	Chateau Langes Co. Ltd.	Clay and sandy soils	The vineyards are located on the plain at the altitude of about 214 m with a cool-warm, semi-humid climate and an annual accumulated temperature of 3,940 °C; with an annual rainfall of about 700 mm and an annual sunshine time of 2,600–2,800 h.
Shacheng of Hebei, (HBSC)	Chateau Des Champs	Sandy soils	The vineyards are located on the Huaizhuo basin at the altitude of about 450–600 m with a warm, semi-arid climate and a big temperature difference between daytime and night time; with an annual accumulated temperature of 3,532 °C and an annual rainfall of about 413 mm.

The original “Cabernet Sauvignon” and “Merlot” grape berries were harvested manually at optimum technological maturity from these regions in 2010, as judged by ratio of sugar and acid content. Pre-fermentation treatments and winemaking were performed as described by Li [35]. Briefly, grapes were crushed on an experimental destemmer-crusher and then transferred to stainless-steel containers. Thirty L of each treatment wine were produced in three replicates. Fifty mg/L of SO_2_ and 30 mg/L of pectinase (Lallzyme Ex) were added to the musts and the contents were mixed by hand. After maceration of the musts for 24 h, 200 mg/L of dried active yeast (*Saccharomyces cerevisiae* strain, Lallemand, Danstar Ferment AG, Switzerland) was added to the musts, according to commercial specifications. Alcoholic fermentation was carried out at 20 to 25 °C to dryness (reducing sugar < 4 g/L) which took place over a 6–8 days period and density controls were maintained during this period. At the end of alcoholic fermentation the wines were separated from pomace, and then added 50 mg/L of SO_2_. After fermentation, the wine samples were bottled and stored at 10–15 °C prior to analysis. All the samples were five months old at the time of analysis. Residual sugar, total acidity, pH, total tannins and ethanol were analyzed [36] (Table 6).

### 3.2. Reagents, Solvents and Standards

Folin-Ciocalteu’s phenol reagent, gallic acid, catechin, *p*-dimethylaminocinnamaldehyde (DMACA), nicotinamide adenine dinucleotide (NADH), phenazine methosulfate (PMS), nitroblue tetrazolium (NBT), 6-hydroxy-2,5,7,8-tetramethylchroman-2-carboxylic acid (Trolox), 2,2-diphenyl-1-picrylhydrazyl (DPPH•) and neocuproine free base were purchased from Sigma-Aldrich (St. Louis, MO, USA). Tris (base) was purchased from Sanland Chemical Co., Ltd. (Los Angeles, CA, USA). The standards, catechin, quercetin, gallic aid, caffeic acid, and resveratrol, were all purchased from Sigma Chemical Co. (St. Louis, MI, USA). Methanol (HPLC grade) was obtained from Fisher Co. (Fairlawn, NJ, USA). All other chemicals and solvents were analytical reagent grade and purchased in China.

### 3.3. Determination of Phenolic Compounds

The total phenols (TP) content was determined by the Folin-Ciocalteu colorimetric method with slight modification [37]. Briefly, an aliquot of 0.1 mL of sample solution (with appropriate dilution if necessary) was mixed with 0.5 mL of Folin-Ciocalteu reagent and allowed to react at 30 °C for 5 min in the dark. Then 1.5 mL of saturated Na_2_CO_3_ solution was added and the mixture was allowed to stand for 2 h before the absorbance of the reaction mixture was read at 765 nm. The total phenols concentration was expressed as gallic acid equivalent.

The total flavonoids (TFO) and the total flavanols (TFA) contents were separately measured according to a colorimetric assay [38] and the DMACA method [39,40], both of them were expressed as catechin equivalent. The total anthocyanins (TA) content was determined by the pH-differential method [41] using two buffer systems–potassium chloride buffer, pH 1.0 (0.025 M) and sodium acetate buffer, pH 4.5 (0.4 M), its content was calculated as malvidin-3-*O*-glucoside.

**Table 6:** General composition of Cabernet Sauvignon and Merlot berries and wines from four different wine grape-growing regions.

Analytical Index	Cabernet Sauvignon								Merlot							
	NXYQY		SXXN		HBCL		HBSC		NXYQY		SXXN		HBCL		HBSC	
	Must	Wine	Must	Wine	Must	Wine	Must	Wine	Must	Wine	Must	Wine	Must	Wine	Must	Wine
Total sugar	217.5 ± 1.4	—	198.6 ± 2.0	—	224.1 ± 1.2	—	204.3 ± 2.0	—	215.0 ± 1.5	—	218.9 ± 1.6	—	205.3 ± 0.8	—	187.3 ± 0.1	—
Titratable acidity (g/L) ^a^	8.1 ± 0.5	7.3 ± 0.1	11.9 ± 0.7	7.1 ± 0.3	7.6 ± 0.3	6.3 ± 0.5	9.3 ± 0.5	6.7 ± 0.2	7.7 ± 0.2	8.0 ± 0.0	8.3 ± 0.4	6.7 ± 0.4	8.9 ± 0.8	6.7 ± 0.1	6.9 ± 0.1	8.3 ± 0.3
pH	3.2 ± 0.1	3.1 ± 0.0	3.1 ± 0.1	3.1 ± 0.1	3.5 ± 0.0	3.6 ± 0.1	3.4 ± 0.2	3.5 ± 0.2	3.0 ± 0.0	3.0 ± 0.0	3.3 ± 0.2	3.3 ± 0.2	3.3 ± 0.1	3.6 ± 0.1	3.0 ± 0.1	3.5 ± 0.1
Total tannins (g/L) ^b^	5.3 ± 0.3	2.5 ± 0.1	3.8 ± 0.1	1.3 ± 0.1	2.3 ± 0.1	2.3 ± 0.2	3.5 ± 0.2	1.5 ± 0.0	2.9 ± 0.1	1.9 ± 0.1	2.6 ± 0.2	1.3 ± 0.1	2.3 ± 0.0	1.6 ± 0.2	2.1 ± 0.1	1.6 ± 0.1
Alcohol (%)	—	12.0 ± 0.2	—	11.0 ± 0.2	—	12.2 ± 0.1	—	13.4 ± 0.2	—	11.0 ± 0.1	—	10.8 ± 0.0	—	11.0 ± 0.1	—	11.5 ± 0.2
Residual sugar (g/L)	—	2.0 ± 0.1	—	2.1 ± 0.0	—	2.8 ± 0.2	—	2.9 ± 0.1	—	1.9 ± 0.0	—	2.1 ± 0.1	—	2.9 ± 0.3	—	3.1 ± 0.3

The results were expressed as mean values ± SD of triplicate samples; ^a^ Values expressed as grams of tartaric acid equivalents per liter basis (g/L); ^b^ Values expressed as milligrams of tannin acid equivalents per liter basis (g/L).

### 3.4. Extraction and Analyses of Individual Phenolic Compounds

#### 3.4.1. Preparation of Sample

The individual phenolics (including flavan-3-ols, flavonols, hydroxybenzoic acids, hydroxycinnamic acids, and stilbenes), a wine sample with 100 mL was diluted with the same volume of distilled water, and then extracted thrice with ethyl acetate (80 mL). The combined ethyl acetate phase was removed by a rotary evaporation at 28 °C and the remainder was resolved in methanol (chromatography grade) up to a final volume of 5 mL. The final sample was filtered by 0.22 μm organic membranes prior to analysis by HPLC-MS/MS.

#### 3.4.2. Quantitative Analyses by HPLC-MS/MS

Qualitative and quantitative analyses of individual phenolic compounds were performed in Center for Viticulture and Enology, College of Food Science & Engineering, China Agricultural University. Based on the optimum HPLC-ESI-MS/MS conditions established for the individual phenolic compounds using standard solutions we further combined retention time with (molecular and fragment) ions information to qualitatively analyze these individual phenolic compounds detected [42]. For the individual phenolic compounds, an Agilent 1200 series instrument, equipped with a G1322A Degasser, a G1312B Binary pump, a G1367C HiP-ALS, a G1316B TCC, a G1314C VWD, and a ZORBAX SB-C18 column (3 × 50 mm, 1.8 μm) were used. The mobile phase comprised of (A) 10% (v/v) acetic acid, and (B) 90% (v/v) acetonitrile containing 10% (v/v) acetic acid. The elution gradient was from 5% to 8% B for 5 min, from 8% to 12% B for 2 min, from 12% to 18% for 5 min, from 18% to 22% for 5 min, from 22% to 35% for 2 min, from 35% to 100%B for 2 min, 100% B for 4 min and from 100% to 5% B for 2 min, with a flow rate at 1.0 mL/min. The injection volume was 2 μL, and the detection wavelength was 280 nm. The column temperature was 25 °C. The MS conditions were as follows: ESI, negative ion model; nebulizer, 35 psi; dry gas flow, 10 L/min; dry gas temperature, 325 °C; scan, 100–1,000 *m/z*.

#### 3.4.3. Quantification of Individual Phenolic Compounds

Flavanols, flavonol, hydroxybenzoic acid, hydroxycinnamic acids, and stilbenes were quantified using catechin, quercetin, gallic acid, caffeic acid, and resveratrol as standards, respectively. The gradience with eight concentrations of mixture standard (catechin, quercetin, gallic acid, caffeic acid, and resveratrol) were set with three replications, five groups of standard curves of the concentration were made based on the average area of individual compounds, and five polyphenol compounds were qualified by the external standard method.

### 3.5. Determination of Antioxidant Activity

#### 3.5.1. Free Radical-Scavenging Activity on DPPH

The ability to scavenge DPPH• free radicals was determined. Scavenging activity was based on the slightly modified method [43]. Briefly, 0.1 mL of sample solution (with appropriate dilution if necessary) was added to 3.9 mL of a 60 μM solution of DPPH• in methanol. A control sample, containing the same volume of solvent in place of extract, was used to measure the maximum DPPH• absorbance. After the reaction was allowed to take place in the dark for 30 min, the absorbance at 515 nm was recorded to determine the concentration of remaining DPPH•. Results were expressed as Trolox equivalent antioxidant capacity.

#### 3.5.2. Cupric Reducing Antioxidant Capacity (CUPRAC)

The cupric reducing antioxidant capacity was determined according to the method of Apak *et al.* [44]. Results were expressed as Trolox equivalent antioxidant capacity.

#### 3.5.3. Superoxide Radical-Scavenging Activity (SRSA)

The method used by Robak and Gryglewski [45] for determination of superoxide anion scavenging activity was followed after some modification. The superoxide radical was generated in 3 mL of Tris-HCl buffer (20 mM, pH 8.3) containing 1 mL of NBT (150 μM) solution, 1 mL of NADH (468 μM) solution and 1 mL of sample solution (with appropriate dilution if necessary). The reaction was started by adding 1 mL of PMS solution (60 μM) to the mixture. The reaction mixture was incubated at 25 °C for 5 min, and the absorbance was measured at 560 nm against the corresponding blank solution. The result was expressed as inhibition in relation to a control test.

### 3.6. Statistical Analyses

The analysis on the same sample was made in three replications and the results were expressed as mean value ± standard deviation. Correlation was calculated by linear regression (SPSS 16.0 for Windows).

## 4. Conclusions

It was verified that the contents of phenolic compounds and antioxidant activities in Cabernet Sauvignon and Merlot wines from the Yuquanling region of Ningxia, were significantly higher than those of the other three regions tested, the next was wines from Shacheng region of Hebei and these parameters in Cabernet Sauvignon and Merlot wines from Changli region of Hebei and Xiangning region of Shanxi were the lowest. The amounts of phenolic materials and antioxidant activity varied considerably in these regional wines, depending on the grape variety and environmental factors of vine growth. Meanwhile, a close relationship between phenolic subclasses and antioxidant activity for all the wine samples was observed. On the basis of our results, differences in measurements of antioxidant capacity determined by alternative methods emphasized the importance of using several methods to assess this parameter, in order to obtain accurate data and to improve the comparison with other data reported in the literature. On the other hand, their composition and content of individual phenolic compounds detected in these regional Cabernet Sauvignon and Merlot wines existed different discrepancies, among them, individual phenolic compounds (catechin, epicatechin, cinnamic acid, quercetin-3-*O*-glucuronide, quercetin-3-*O*-glucoside, laricitrin-3-*O*-glucoside and isorhamnetin-3-*O*-glucoside) revealed a significant correlation (*p* < 0.05) with the antioxidant capacity estimated by the three methods (significant correlation with at least one of the methods), especially for catechin and epicatechin, and thus they seem to be the major contributor to antioxidant capacity.
